# Dynamic progression of the calf’s microbiome and its influence on host health

**DOI:** 10.1016/j.csbj.2021.01.035

**Published:** 2021-01-26

**Authors:** Nida Amin, Jana Seifert

**Affiliations:** Institute of Animal Science, University of Hohenheim, Stuttgart, Germany

**Keywords:** Calve, Microbiome, Gastrointestinal tract, Feeding, Host health, Immune system

## Abstract

The first year of a calf’s life is a critical phase as its digestive system and immunity are underdeveloped. A high level of stress caused by separation from mothers, transportation, antibiotic treatments, dietary shifts, and weaning can have long-lasting health effects, which can reduce future production parameters, such as milk yield and reproduction, or even increase the mortality of calves. The early succession of microbes throughout the gastrointestinal tract of neonatal calves follows a sequential pattern of colonisation and is greatly influenced by their physiological state, age, diet, and environmental factors; this leads to the establishment of region- and site-specific microbial communities. This review summarises the current information on the various potential factors that may affect the early life microbial colonisation pattern in the gastrointestinal tract of calves. The possible role of host–microbe interactions in the development and maturation of host gut, immune system, and health are described. Additionally, the possibility of improving the health of calves through gut microbiome modulation and using antimicrobial alternatives is discussed. Finally, the trends, challenges, and limitations of the current research are summarised and prospective directions for future studies are highlighted.

## 1 Introduction

The development of the gastrointestinal tract (GIT) in neonatal humans and animals is a highly dynamic process that is influenced by genetic and environmental factors, nutrition, and the concomitant development of the intestinal microbial communities. This is also true for ruminants, where the first month of life is even more challenging as the rumen is less developed. The rumen is the largest forestomach in ruminants and is highly important for the conversion of ingested feed particles into metabolites that are absorbed and utilised by the host and the formation of microbial protein sources used by the animals [1]. Young ruminants are functionally monogastric at birth with an underdeveloped forestomach system, including the rumen, reticulum, and omasum. During these first months of life, the abomasum and intestines serve as their major digestion sites [2]. The establishment of a fully mature system requires the development of the reticulo-rumen and the associated microbiomes [3]. The microbial communities in the rumen follow a sequential pattern of colonisation with bacteria as the first colonisers, followed by the methanogenic archaea, anaerobic fungi, and protozoa [4], [5], [6]. However, studies using molecular-based techniques showed initial rumen colonisation with facultative anaerobic bacteria (*Enterococcus* and *Streptococcus*) in new-born calves as well as archaea within a few hours after birth [7], [8]. A recent study by Malmuthuge et al. (2019) reported on rumen colonisation in neonatal calves with an active bacterial community at birth. The rumen of one-week-old calves were already colonised by active complex-carbohydrate-fermenting bacterial species even in the absence of solid substrates in the diet [9]. These initial gut colonisers utilise the oxygen available in the gut, thus, creating an anaerobic environment favourable for the growth of strict anaerobic gut communities, including *Bifidobacterium* and *Bacteroides* [10], [11]. The strict anaerobic bacterial community, including cellulolytic and proteolytic bacteria, together with niche specialists, establish and dominate the gut microbiome within the first two weeks of life [7], [12], [13], [14].

**Fig. 1:**
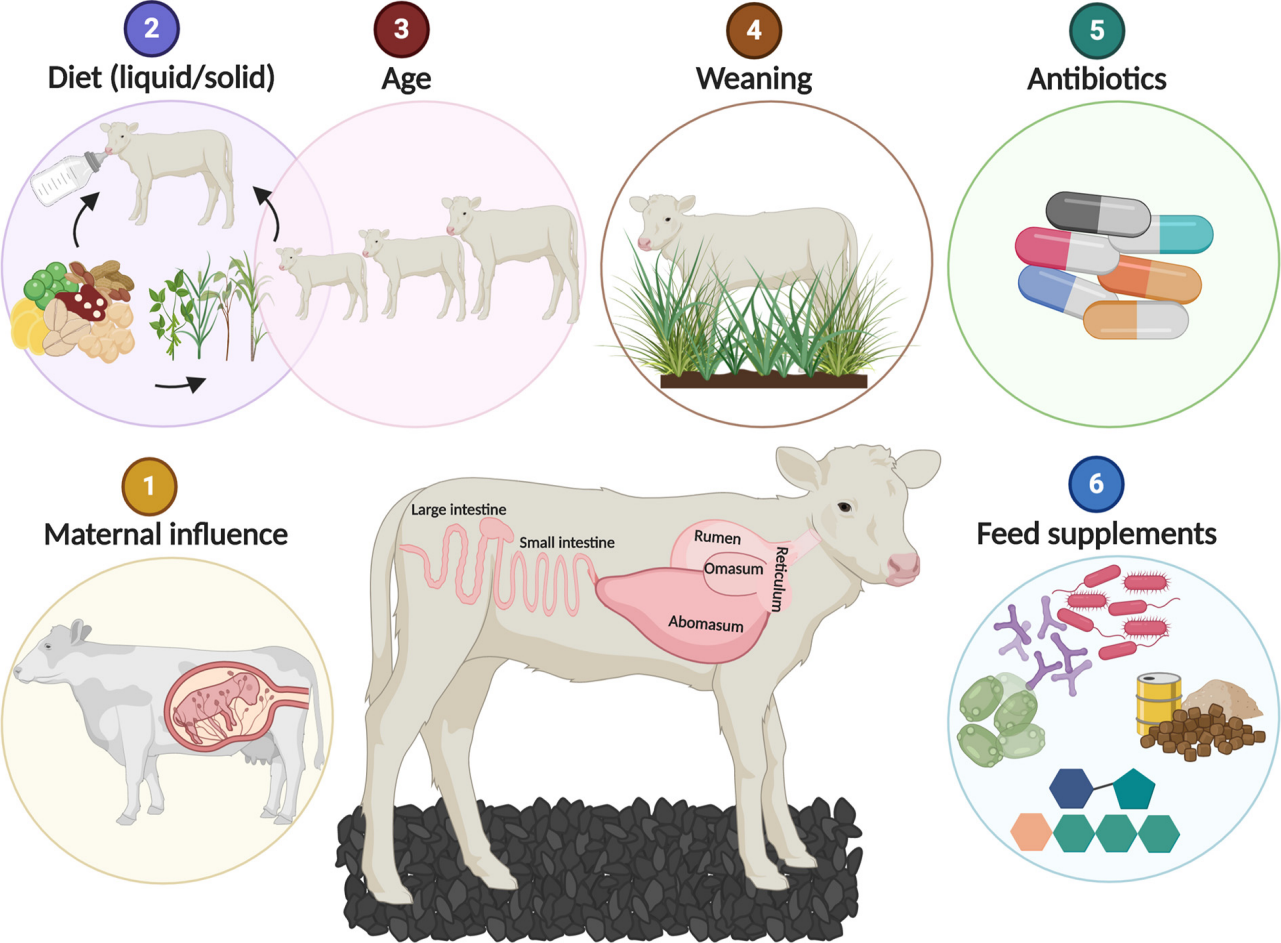
Factors that influence the initial establishment and development of microbial communities throughout the GIT of neonatal calves. Figure created with BioRender.com.

The establishment of a strict anaerobic bacterial community in the GIT of neonates plays an essential role in mucosal immune system development, and is therefore, a critical phase for the host [15], [16]. After the initial gut colonisation, constant exposure of the host GIT to specific microbes is necessary to maintain the host’s energy metabolism, health, and mucosal immune system maturation [17], [18]. Once the GIT is fully mature and the climax microbial community is established, the intestinal microbiome is considered stable thereafter, except for changes in the host’s health, physiological state, and diet [19], [20], [21]. However, considerable differences exist in microbial community profiles in different regions of the GIT in ruminants [14]. Similarly, the mucosa-associated microbial communities were found to differ from those occupying the lumen [14], [22], [23], [24], [25], suggesting a possible role of host–microbe interactions in defining such diverse microbial community structures. In this review, the development of microbial communities across the GIT of calves under the influence of maternal microbiota, age, diet, weaning, and environmental factors (antibiotics and pre/probiotics) (Fig. 1 and Table1), and the possible role of host–microbe interactions in the development of the host’s gut, immunity, and health is summarised.

**Table 1:** Overview of major factors that affect the initial colonization of microbial communities throughout the GIT of neonatal calves, host gut and immune system development.

Sample type	Calf age at the time of sampling^1^	Diet^1^, ^2^	Method^3^	Year	Reference
**MATERNAL INFLUENCE**					
Faeces	0, 6, 12, 24 and 48 h, 3, 7, 14, and 42 days	N.D.	DNA, PCR single strand conformation polymorphism (PCR-SSCP) of V4-V5 region	2012	[28]
	4 days–20 days				
Faeces	24 h and 7 days	Colostrum: 4–6 h after birth, followed by pooled cow milk	DNA, qPCR, V3-V4 amplicon sequencing (Illumina)	2018	[29]
Overall GIT	0, 1, 2, 3, 4, 5, 7, 14, and 21 days	Milk replacer (MR) throughout the study	DNA, V3-V4 amplicon sequencing (Illumina)	2018	[25]
Faeces and mouth	Faeces (0.5, 6,12, 24, and 48 h); mouth (0.5 h)	Colostrum: after 0.5 h till the end of trial	DNA, V3, V4, V5 amplicon sequencing (Illumina)	2019	[30]
**WEANING**					
Rumen and faeces	36 and 54 days	Abrupt weaning: MR until day 48, reduction to 0 within 24 h; Gradual weaning: MR slowly reduced from day 36 to day 49; all calves had ad lib. access to water, starter and chopped straw from day 7 to day 54	DNA, V4 amplicon sequencing (Illumina)	2016	[79]
Rumen	N.D., after weaning	Fresh milk: day 1 to day 7; half fresh milk and half MR until day 13; MR and dry feed starter till the end of trial; starter, grass hay and water were available ad lib.	DNA, qPCR	2017	[80]
Rumen and faeces	5, 7, and 9 weeks	Ad libitum access to water, starter, chopped straw and oat straw from birth till the end of trial	DNA, V4 amplicon sequencing (Illumina)	2017	[84]
**ANTIBIOTICS**					
Rectal swabs and faeces	Newborn, 1, 2, 3, 4, 5, 6, 7, 8, 9, and 10 weeks	Trial 1: Milk substitute without antibiotics or antibiotic containing fresh milk or fermented milk	Culture-based assays	1990	[143]
		Trial 2: Standard milk substitute, containing growth promoter or antibiotic containing milk			
Rectal swabs	N.D.	Colostrum: within 24 h after birth; ad lib. milk with penicillin G and water: until day 37	Culture-based assays	2003	[144]
Faeces	N.D.	Bulk milk (BM) and grain concentrates with or without oxytetracycline: 12 weeks trial	Culture-based assays and PCR for screening of drug resistance genes	2004	[133]
Rectal faecal swabs	0, 2, 4, and 6 weeks	N.D.	Culture-based assays	2005	[145]
Faeces	9 time points during first 6 months	Pasteurized or non-pasteurized waste milk before weaning	Culture-based assays	2012	[146]
Faeces	6, 7, and 12 weeks	Colostrum: within 2–6 h after birth; MR without antibiotics or with neomycin sulfate and oxytetracycline hydrochloride antibiotics; all calves ad lib. access to starter grain from day 1; alfalfa hay offered post-weaning	DNA, qPCR, sequencing of target genes	2012	[150]
Faeces	2, 14, 28, and 56 days	Colostrum: within 2–4 h after birth; ad lib. hay: from day 1; pasteurized or non-pasteurized (WM and BM): from day 3; pelleted calf starter: from day 8 until day 56	Culture-based assays	2013	[132]
Faeces	12 days	MR: from day 0 with or without bacitracin methylene disalicylate. all calves: ad lib. to concentrate from day 3 until day 56	DNA, V4-V6 amplicon sequencing (454)	2013	[137]
Faeces	3, 5, and 6 weeks	Pasteurized hospital milk throughout the study. Water and calf starter ad lib.	DNA, V1-V2 amplicon sequencing (454)	2015	[138]
Faeces	Newborn, 1, 2, 3, 4, 5, and 6 weeks	Colostrum: within 4 h after birth; raw milk without antibiotics or with low concentrations of ampicillin, ceftiofur, penicillin, and oxytetracycline: from day 1 till the end of trial; pelleted calf starter: offered from day 7 until day 42	DNA, V4 amplicon sequencing (Illumina)	2016	[139]
Faecal and nasal swabs	42 days and 1 year	Colostrum: after birth; MR or WM: for 6–12 weeks	Culture-based assays	2017	[147]
Faecal swabs	3, 35, and 56 days	Colostrum: within the 24 h after birth; MR without antimicrobials or pasteurized WM with β-lactam residues: until day 49. all calves ad lib. water and textured calf starter: from day 1 to day 56	Culture-based assays and PCR of antimicrobial resistance genes	2017	[148]
Faeces	0, 1, 3, and 6 weeks	Milk without antimicrobials or with low concentrations of ceftiofur, penicillin, ampicillin and oxytetracycline: birth till 6 weeks of age	DNA, and whole genome sequencing (Illumina)	2018	[151]
Faeces, ileum, colon	35 days	Colostrum: within 1 h after birth; MR without antibiotics or with low concentrations of antibiotics. all calves ad lib. water and starter feed from day 4 until end of trial	DNA, V3-V4 amplicon sequencing (Illumina)	2018	[140]
Faeces	N.D.	Colostrum: within hours after birth; Pasteurized non-saleable milk: until 56 days of age. Ad lib. water	DNA, whole genome sequencing (Illumina)	2019	[141]
Rumen fluid and tissues	15, 25, and 35 days	Colostrum: within 1 h after birth; MR without antibiotics or with low concentrations of penicillin, streptomycin, tetracycline and ceftiofur. all calves ad lib. starter and water from day 2 until end of trial	DNA, V3-V4 amplicon sequencing (Illumina)	2019	[142]
Faeces	N.D.	Colostrum: within 1 h after birth; pasteurized non-saleable milk until 56 days of age. ad lib. water	Culture-based assays and PCR	2020	[149]
**FEED SUPPLEMENTS**					
**Probiotics**					
Faeces	7–35 days	Trial 1: MR without or with *B. pseudolongum* / *L. acidophilus*: from day 7 to day 42 d; starter: from day 14 to day 56. ad lib. water and dried grass	Culture-based assays	1995	[154]
		Trial 2: MR without or with *B. thermophilum, E. faecium* and *L. acidophilus*; ad lib. MR without antibiotics and water			
Rumen contents and faeces	31–33 days	MR until 6 weeks of age, afterwards a mixture of alfalfa pellets and sweet feed with ad lib. water throughout the trial	Culture-based assays and genomic DNA fingerprinting	1998	[157]
Faeces and blood	1, 3, 5, and 7 weeks	Non-pasteurized colostrum: after birth; acidified non-saleable milk: day 1 - day 56. Ad lib. water and calf starter	DNA, V4 amplicon sequencing (Illumina)	2015	[155]
Blood, and tissue and digesta of jejunum, ileum and colon	Blood (1 and 12 h, 1–7 days); Tissue and digesta (1 week)	Colostrum replacer: first 12 h; MR: from day 1 to day 7 with or without supplementation of *Saccharomyces cerevisiae boulardii*. Ad lib. water.	Radial immunodiffusion analysis, ELISA, immunohistochemistry, RNA and DNA, RT-qPCR	2020	[159]
**Prebiotics**					
Rumen fluid and blood	N.D.	Milk and concentrate feed (with or without cellooligosaccharides or kraft pulp supplements): from 4 weeks before weaning till 12- or 16-weeks post-weaning	DNA, qPCR	2019	[161]
Rumen fluid and blood	6.5, 7, 7.5, and 8 months	Ad lib. starter concentrate, chopped oat hay and water: for 1 week; oat hay and concentrate (3:7) with or without astragalus root extract: afterwards	Manual assay for serum; DNA, V3-V4 amplicon sequencing (Illumina)	2020	[162]
**Dietary supplements**					
Rumen	90 and 160 days	Whole milk: first 30 days; MR and starter concentrate (with or without calcium propionate supplement): day 30 to day 90; starter feed: day 91 till the end of trial; alfalfa hay was only provided at day 91.	DNA, V4 (bacteria) and V8 (archaea) amplicon sequencing (Illumina)	2020	[164]
Faeces and blood	Faeces (1, 3, 7, and 14 days); Blood (14 days)	Colostrum: within 1 h after birth; Raw milk: day 2 to day 4; starter concentrate (with or without zinc supplement): day 4 till the end of trial	ELISA, DNA, V3-V4 amplicon sequencing (Illumina)	2020	[163]
**HOST IMMUNE SYSTEM DEVELOPMENT**					
Ileum and colon tissues, plasma, adrenal glands	Plasma samples (72 h); other (75 h)	Colostrum: immediately after birth. three groups: a) colostrum, b) whole milk, c) mixture of 50% colostrum and 50% whole milk: for 72 h	RNA, qRT-PCR and qPCR	2020	[113]
Jejunal mucosa	80 days	Colostrum: immediately after birth; acidified transition milk: first 3 days; MR: day 4 until 8 weeks of age with linear reduced amount during week 9 to 10. Ad lib. water, hay and concentrate from day 10	RNA, Illumina HiSeq sequencing	2018	[115]
Blood, jejunum mucosa	Blood (1, 2, 7, 14, 21, 28, 35, 42, 49, 56, 63, 70, and 77 days) Jejunum (day 80)	Colostrum: within 2 h after birth; acidified transition milk until day 3; MR: day 4 until day 70. Ad lib. water, hay and concentrate from day 10	RNA, whole transcriptome sequencing	2018	[117]
Rumen, jejunum, ileum, cecum, and colon	3 weeks	Fresh whole milk and calf supplement throughout the trial	DNA, V1-V3 amplicon sequencing (454), qPCR	2014	[14]
Mucosa of rumen, jejunum, ileum, cecum and colon	3 weeks and 6 months	Non-pasteurized whole milk and calf supplement: first 12 weeks; alfalfa hay and oats: for the next 4 months	DNA, fingerprinting, clone libraries, qPCR	2012	[119]
**HOST GUT DEVELOPMENT**					
Rumen, jejunum and ileum tissues	Newborn, 7, 21, and 42 days	Colostrum: after 30 min. of birth; whole milk: until day 7; ad lib. starter: from day 7 until day 42	DNA and RNA; Illumina RNA-sequencing and qRT-PCR	2014	[122]
Rumen tissue and content	Newborn, 1, 3, and 6 weeks	Colostrum: within the first 3 days; whole milk: day 4 till the end of trial. Ad lib. starter from second week of life	DNA, whole genome sequencing (Illumina), qPCR, RNA, transcriptome (host)	2019	[9]

1 N.D. = Not defined.

2 Ad lib. = ad libitum.

3 Hypervariable regions (V1, V2, V3, V4, V5 V6 and V8) of prokaryotic 16S rRNA gene.

## 2 Early succession of microbes throughout the GIT of neonatal calves and maternal influence

Birth exposes neonates to the vaginal, skin, and colostrum microbiome of the mother [26], [27], which initiates the microbial colonisation of the neonatal GIT. The neonatal microbiome must undergo several modifications prior to weaning (6–12 weeks), and it may take a year for the establishment of a fully functional and stable GIT microbial community [7]. To date, only a few culture-independent studies have examined the effect of maternal sources on the early establishment of microbes in neonatal calves’ GIT [25], [28], [29], [30]. At the genus level, the rectal microbiota of the new-born calves was more similar to the dam’s oral microbiota (39%) as compared to the microbiota on the dam’s vagina (24%) or faeces (15%), indicating an *in utero* transfer route for the inoculation of neonatal gut microbiota [29]. However, the faecal microbiota during the first 48 h of calf life showed a close resemblance to the dam’s vaginal microbiota than other maternal sources (faeces or colostrum), indicating the possible transfer of microbes to the neonates via the birth canal [30]. In contrast, Yeoman et al. reported high similarity between the dam’s udder skin and calf’s GIT microbiota during the first three weeks of life [25]. The inconsistencies among these studies are probably due to differences in sampling sites (calf faeces *vs.* dam’s mouth, vagina, faeces, udder skin, or colostrum), and sampling time. In addition to the influence of maternal interaction/microbiome on the early succession of microbes throughout the neonatal calves’ GIT, the facility, farm or location where the calves are born and raised also reported to have a significant impact on the gut microbiota of Holstein dairy cows [31] as well as beef calves [32], [33]. Thus, the management practices must be carefully considered because of their unidentified role in shaping gut microbial community structures besides several other factors including genetics, breed, age, diet and study method etc.

## 3 Effect of early feeding regimen and age on the initial establishment and development of microbial communities in the GIT of neonatal calves

Young ruminants are pseudo-monogastric at birth with an underdeveloped reticulo-rumen, relying solely on a milk-based diet [2]. In pre-weaned calves, most of the liquid feed flows straight into the abomasum without entering the rumen; thus, the small and large intestines serve as their major digestion sites. The forestomach system in neonatal calves changes tremendously during the first year of life, with a shift in the activity of intestinal enzymes (lactase and maltase), which facilitates the development of the salivary apparatus, other digestive compartments, and rumination behaviour in calves [34], [35], [36]. In addition, rumen volume increases, and rumen papillary shape and size proliferate, providing better niche environments for the microbial colonisation of the rumen and its subsequent functioning [37]. Concomitant with these morphophysiological adaptations, the changes in microbial composition of pre-weaned calves’ GIT are driven by the rearing environment, age, and diet [17], [33], [36], [38], [39]. The diet of pre-weaned calves is changed gradually from milk or milk replacer (MR)-based diets to solid feed within the first few weeks of their lives [40]. These dietary shifts seem to have prominent effects on the neonatal calf microbiome. Many studies have explored the effect of liquid/solid diets, including fresh or heated colostrum [41], [42], whole milk, waste milk (WM), pasteurised waste milk (pWM) or MR [43], [44], [45], starter concentrate [23], [46], [47] and roughage [48], [49], [50], [51], on the initial establishment of bacterial communities in the GIT of neonatal calves.

### 3.1 Colostrum and other liquid feeds

New-born calves are immunodeficient and depend solely on colostrum-associated immunoglobulins [52]. Feeding high-quality colostrum is highly recommended as it can inhibit the growth of pathogens, stimulate the colonisation of the small intestines with beneficial microorganisms [41], increase body weight gain, improve the development and function of the GIT, reduce the risk of diarrhoea [53] and thereby, decrease the mortality rate in calves [54]. However, the lack of proper hygiene practises increases the risk of colostrum contamination with microbes [55]; therefore, adequate heating of colostrum is recommended. Feeding heat-treated colostrum within the first 12 h of life inhibited pathogenic *Escherichia coli* and *Shigella*, and increased the growth of *Bifidobacterium* [41], [42]. The increase in *Bifidobacterium* was also observed in 51-hour-old dairy calves using a similar treatment [56].

After colostrum feeding, the nutrient composition of the subsequent feeding again defines the microbiome composition. In general, the rumen bacterial community of one- to three-day-old colostrum-fed calves was dominated by Proteobacteria [7], [57], but as the calves aged and started to consume MR and starter concentrate-based diet, Proteobacteria was slowly replaced by Bacteroidetes in the rumen [7], [12], [57]. Similar to the rumen, Proteobacteria dominated the faecal microbiota of 24–48-hour-old calves, showing a depletion and a subsequent increase in Firmicutes within the first seven days of a calf’s life without any diet change [29], [30]. Similarly, Firmicutes was the dominant phylum in the faecal microbiota of one- to seven-week-old calves [13]. Yeoman et al. also reported higher abundance of Firmicutes in the colon and faeces, while Bacteroidetes was more abundant in the rumen, reticulum, omasum, and abomasum within the first three weeks of a calf’s life [25].

Shifting the diet of pre-weaned calves (7–28 days) from colostrum to whole milk increased the abundance of typical milk-utilising bacteria (*Lactobacillus, Parabacteroides*, and *Bacteroides*) in their rumen [47]. Feeding milk to two-week-old calves also increased the abundance of *Ruminococcus flavefaciens*, a fibrolytic bacterium in the rumen [46]. Similarly, a recent study by Malmuthuge et al. reported the colonisation of a whole milk-fed one-week-old calf’s rumen with active *R. flavefaciens*, whose density increased with increasing age, suggesting the possible use of milk as a substrate for *R. flavefaciens* [9]. Feeding a milk-based diet also had prominent effects on the lower gut microbiota of pre-weaned calves as indicated by the high levels of the *Bacteroides–Prevotella* group and *Faecalibacterium* in the faecal samples of MR-fed one-week-old calves [58]. Similar levels were also reported in the colon samples of three-week-old whole milk-fed calves [14], indicating that faecal samples represent the microbiome of the large intestine in an adequate manner [14]. Similarly, Alipour et al. also observed a high dominance of *Faecalibacterium* and *Bacteroides* in the faecal samples of seven-day-old milk-fed calves [29].

The cost benefits of WM over whole milk and MR [59], [60] and the increased use of on-farm pasteurisers have facilitated the use of waste milk in calf feeding programmes. Feeding WM modified the rumen bacterial community composition by decreasing *Prevotella* 7 and increasing *Butyrivibrio* 2, the *Rikenellaceae* RC9 gut group, and *Prevotellaceae* UCG-003 in two-month-old calves [45]. The opposite was true when WM feeding was prolonged during the first six months, and higher abundance of *Prevotella* 7 and *Succinvibrionaceae* UCG-001 and lower abundance of *Prevotellaceae* UCG-003, *Rikenellaceae* RC9 gut group, *Selenomonas* 1, and others were observed [45]. Pasteurisation inactivates the vegetative bacterial cells, reduces the risk of disease transmission and mortality and improves the growth rate of calves [61]. A relatively high abundance of *Prevotella* and low abundance of *Streptococcus* and *Histophilus* were observed in the nasal microbiota of pWM-fed 42-day-old calves [44]. In addition, feeding pWM increased faecal bacterial diversity from two weeks to six months of age; a higher prevalence of faecal Bacteroidetes and lower prevalence of Firmicutes, and no *Salmonella* were detected in young pWM-fed calves [43]. An opposite, but non-significant, ratio of Firmicutes and Bacteroidetes was also found in pWM-fed calves as compared to that in MR-fed calves [44]**.** The effects of MR-compositions on faecal microbial communities were studied recently, and it was found that the faecal microbiota of seven-day-old calves fed with MR enriched with conjugated milk oligosaccharides had higher relative abundance of *Faecalibacterium prausnitzii and Bifidobacterium* species than did those consuming MR with high free milk oligosaccharides [62]. *F. prausnitzii* is a beneficial bacterium for neonatal calves due to its positive correlation with body weight gain and reduced diarrhoea [13]. Furthermore, Yak calves reared in isolation on a standard MR-, starter concentrate-, and hay-based diet were found to have better organ development, growth rate, immune function, and higher abundance of non-fibrous carbohydrate-utilising bacterial genera [63] than the maternally nursed and grazed calves that had a higher abundance of fibrous carbohydrate-utilising bacterial genera [63], [64]. Thus, the early feeding regimen shapes the microbiome structure in pre-weaned calves by providing different substrates for growth and establishment of various ecological niches.

In addition, drinking water offered to the calves immediately after birth seems to have a prominent impact on gut microbial composition, as indicated by the increased abundances of *Faecalibacterium* and *Bacteroides* at two weeks and *Faecalibacterium* and *Bifidobacterium* in the six-week-old calves [65]. In addition, calves consuming drinking water from birth had higher body weight, digestibility of fibre, and feed efficiency than the calves that started to receive drinking water from 17 days of age [66].

### 3.2 Consumption of solid feed reshapes the gut microbiota in pre-weaned calves

The solid feed intake begins around two to three weeks of life, which initiates the critical transition process leading to the establishment of a fully functional rumen. It is usually characterised by a constant or gradual supply of concentrate and ad-libitum hay in addition to milk feeding. Thus, the effects of solid feed intake should be considered as complex responses to enhanced starch-rich and moderate fibrous feed ingredients together. Generally, an increased abundance of amylolytic and fibrolytic bacteria, such as *Succinovibrionaceae*, *Fibrobacteraceae*, and *Prevotellaceae*, in the rumen microbiome has been described in almost all studies of this feeding period [7], [46], [48], [49], [50], [57], [67], [68], [69], [70]. *Prevotellaceae* is the predominant family in the rumen fluid and has a broad genetic capacity to use a variety of soluble sugars, starch, protein, and peptides [71], [72], [73]. The enzymes involved include carbohydrate-degrading enzymes (CAZYmes), such as glycoside hydrolases (GH2, GH3, GH42, and GH92), which are detectable in pre-ruminant rumen samples [12]. The activity of amylase and xylanase has already been shown in two-day-old calves, even in the absence of complex dietary carbohydrates [74]. Thus, the presence of glycoside hydrolase activity together with the production of short-chain fatty acids (SCFA) reveals that the metabolically active rumen microbiome is established soon after birth in neonatal calves, even in the absence of solid feed. SCFA are important for rumen tissue metabolism, rumen papillae, and epithelium development [9], [40] and they are absorbed into the bloodstream through the papillae and provide energy for calf metabolism and growth [40]. Depending on the solid feed source, changes in the pH and SCFA amount and composition are observed. Forage feeding improves the ruminal environment by increasing rumen liquid pH [40], [48], reducing the chances of subacute ruminal acidosis, and modifying the structure of the rumen microbiome, leading to the establishment of a fully functional rumen during weaning [49], [75]. Furthermore, the particle size as well as the physical form of diet seems to influence the morphophysiological and microbial development of the rumen [76], [77]. Feeding a ground diet to calves reduces the growth of their rumen papillae, lowers the pH of their rumen liquid, reduces the number of cellulose-degrading bacteria, and increases the number of amylose degraders [76]. This finding strongly indicates the potential role of effective fibre feeding for the modification of the rumen environment as well as the associated microbial community composition.

The establishment of an archaeal community in the GIT of calf is important for the required hydrogen balance during bacterial fermentation. The dietary modifications also seemed to have obvious effects, and a higher abundance of *Methanosphaera* and lower abundance of *Methanobrevibacter* were observed in the rumen of pre-weaned calves fed a milk plus starter concentrate-based diet as compared to the milk-fed calves [47]. Starter concentrate feeding also increased the dominance of *Methanomicrobiales mobile* in the abomasum, caecum*,* and faeces and *Methanobrevibacter* in the caecum and faeces of 20-day-old calves, as well as decreasing the abundance of *Methanococcales votae* [46]. Additionally, a decrease in the rumen bacterial diversity, and an increase in the rumen archaeal diversity as well as fungal richness were observed with silage supplementation [51]**.**

## 4 Effect of weaning age and management on microbial colonisation of the GIT in calves

Among the most important factors influencing further animal development in general, and the forestomach system in particular, are the date (age) and strategy of weaning. The abrupt weaning of calves from a milk-based diet to the consumption of solid feed decreases their solid feed intake and average daily gain [40], [78]. However, no effect of the weaning strategy (abrupt *vs.* gradual) was observed on the establishment of rumen and faecal microbial community composition [79], suggesting that the progressive development of the microbial community into a mature state occurs with age [12]. The date of weaning is an important factor in the development of the rumen. Weaning calves, at eight weeks of age, increased their average daily gain [80]**,** and improved carcass quality, feedlot growth, and performance [81], [82]. Rumen enzyme activity was also improved [80], probably due to a greater concentrate intake [83], indicating that the consumption of solid feed triggers the development of the adult-like rumen bacterial community. However, calves weaned six weeks after birth abruptly shifted the β-diversity of their rumen and faecal microbiomes compared to the calves weaned eight weeks after birth [84].This sudden change in the microbial community structure of early weaned calves reflects pre-mature rumen development, paralleled by their reduced growth rate [85], whereas gradual rumen development, [84] improved feed intake, and growth rates were observed when calves were weaned at eight weeks of age [85]. Thus, a balanced weaning management and an appropriate weaning age are important to minimise the side effects.

Rumen fermentation activity begins with the addition of solid feed in the diet and concomitantly alters the microbial composition of a calf’s GIT. An increase in the abundance of Firmicutes and Proteobacteria and a decrease in the abundance of Bacteroidetes were observed in the rumen microbial community from pre- to post-weaned state [79]. Bacteroidetes dominated the rumen microbiota of 42-day-old [12] and two-month-old pre-weaned calves [7]. A similar weaning-related decrease in the abundance of Bacteroidetes and a subsequent increase in Firmicutes were observed, regardless of the calf’s age at weaning [84]. This suggests that the rumen of pre-weaned calves contains the same dominant phyla, including Bacteroidetes, Firmicutes, and Proteobacteria, as found in the rumen of mature post-weaned calves, although the abundance of these phyla varies depending on the developmental stage [86]. At the genus level, *Prevotella* dominated the rumen microbial community of both pre- and post-weaned calves and showed no changes in the abundance regardless of weaning age or strategy [79], [84]. Similarly, high dominance of *Prevotella* in the mature rumen of two-month- to two-year-old cattle has previously been reported [7], [12] Nevertheless, the genus level composition of MR-fed pre-weaned calves’ rumen showed a higher relative abundance of *Bacteroides* and *Succinivibrio* than did that of post-weaned calves fed a high-starch diet [79]. In contrast to this depletion, the abundance of *Sharpea* increased by weaning, making it the second dominant genus in the rumen of post-weaned calves [79]. The increase in starter and forage intake from pre- to post-weaned period [79], [87] was positively correlated with the calf’s body weight and the abundance of *Sharpea* [79]**.** However, the abundances of *Shuttleworthia* and *Dialister* increased drastically in early weaned calves across weaning, while no differences were observed in late-weaned calves before and after weaning [84]. *Dialister* spp. are capable of degrading starch [88], and the increased abundance of *Dialister* in early weaned calves was probably due to increased consumption of starter concentrate across weaning [84]. In addition, early weaned calves had higher number of *Fibrobacter succinogenes* and *Ruminococcus albus*, with *a lower* number of *Butyrivibrio fibrisolvens,* than did late-weaned calves [80]. *Ruminococcus* abundance was positively correlated with solid feed intake and body weight gain in calves [79], likely reflecting the cellulolytic capabilities of *Ruminococcus* species, which are found in the mature rumen [7], [89]. Therefore, it can be speculated that as soon as the calf started to consume the solid feed, the bacterial community resembling the mature rumen is established.

Contrary to the bacterial community of the rumen, the faecal bacterial community of pre-weaned calves showed a high dominance of Firmicutes being replaced by Bacteroidetes in post-weaned calves [79]. At the genus level, the abundance of faecal *Bacteroides* decreased due to weaning, but it remained the predominant genus in both the pre- and post-weaned state [79]. Furthermore, an increase in the abundance of *Prevotella* was observed due to weaning [79]. However, the abundances of the major faecal bacteria remained unaffected by weaning [84]. Nevertheless, an increased abundance of *Ruminococcus* and a decreased abundance of *Blautia* were observed in post-weaned calf faeces [79], [84], likely reflecting a shift from intestinal to ruminal fermentation in post-weaned calves.

Rumen carbohydrate metabolism showed an age-dependent increase between 5 and 9 weeks, regardless of weaning. Conversely, a decline in faecal carbohydrate metabolism was observed from the pre- to post-weaned state [84]. Additionally, a decrease in rumen bacterial diversity and evenness and an increase in faecal bacterial diversity, richness, and evenness were observed in post-weaned calves [79]. This was probably due to the higher solid feed intake in the post-weaning period, resulting in a greater amount of substrates reaching the lower intestine [79]. Thus, the higher substrate availability and lower pH variability of the hindgut favoured higher bacterial diversity in the lower digestive tract of ruminants.

## 5 Distinct bacterial communities are associated with the mucosal epithelium and luminal digesta of the GIT of calves

The bacterial composition in the GIT of animals and humans varies among the gut regions, with considerable differences between the microbes associated with the epithelial mucosa and those occupying the luminal digesta. This is also true for calves [14], [22], [23], [24], [25] and adult ruminants [90]. The mucosa-associated microbial community in calves is found to have higher individual variation, diversity, richness, and a lower microbial load than the microbiota in digesta samples [17], [22], [23], [24], [90]. These differences are caused by variations in host physiological state and immunity, interactions between the symbiotic bacteria and host epithelium, pH, oxygen gradient, nutrient profile, and dietary transition rates [91], [92]. Each of these factors defines the microbial colonisation potential of each site, thus resulting in site- and region-specific microbial community establishment.

Digesta-associated gut communities within the first 21 days of a calf’s life, except for the colon, showed a high dominance of Firmicutes [14], [25], whereas a higher abundance of Bacteroidetes was observed in the mucosa-associated communities, except jejunal tissues, suggesting that the early life mucosal environment favours the colonisation by Bacteroidetes than Firmicutes [14]. Proteobacteria were also more abundant in the mucosa than the digesta samples [14], [25], suggesting that the mucosa-associated Proteobacteria spp. might play an essential role in scavenging blood oxygen and ruminal ammonia oxidation [14]. This would promote an anaerobic environment for the colonisation and fermentative activities of rumen microorganisms [14]. Such compositional changes in the mucosa or digesta-associated communities were more prominent at the genus level, where *Bacteroides* dominated the digesta-associated communities in the reticulum, rumen, omasum, abomasum, caecum, and colon [14], [25]. In contrast, the mucosa-associated bacterial communities of the rumen, ileum, caecum, and colon were dominated by *Prevotella* [14]. Moreover, the abundance of *Escherichia* exceeded *Bacteroides* in the mucosal samples of the omasum, abomasum, ileum, colon, and faeces [25]. Similar to this study, the hindgut microbiota of one-week-old calves showed a high dominance of mucosa-associated *Escherichia-Shigella* groups, indicating greater disease susceptibility in young calves [24]. The digesta-associated community of the duodenum was dominated by *Lactobacillus*, while *Pseudomonas* dominated in the mucosa [25]. Furthermore, the mucosa-associated communities of the jejunum showed high abundances of *Prevotella, Pseudomonas, Acinetobacter, Rikenellaceae* RC9 group*,* and *Delftia* [25]. The high dominance of aerobic/facultative anaerobic bacteria (*Pseudomonas, Acinetobacter, Delftia*, and *Escherichia)* in several mucosal samples suggests that these bacteria prefer gastrointestinal epithelium for growth due to higher availability of oxygen concentration [93]. In contrast, the jejunal digesta-associated communities were dominated by *Sharpea, Butyrivibrio, Ruminococcus*, *Lactobacillus* [14], *Streptococcus*, and *Escherichia* [25]. *Sharpea* spp. are capable of fermenting a vast variety of sugars [94]. Their high dominance in jejunal digesta of three-week-old calves is indicative of their important role in the fermentation of milk during early calf life [14].

The mucosa-associated bacterial community composition was also affected by calves’ age and was significantly correlated with SCFA concentrations, indicating that the host physiology as well as diet play a role in shaping mucosal microbial communities [24]. The abundance of mucosa-associated *Escherichia-Shigella* was negatively correlated with acetate concentration and the inhibition of *E. coli* growth was observed due to high concentration of acetate [95]. SCFA can also influence the turnover of intestinal epithelial cells [96], indicating a possible interaction between mucosa-associated microbial communities and digesta-associated microbial metabolites [24].

## 6 Influence of host genetics on gut microbial colonisation and systemic immunity in neonatal calves

In recent years, many studies have evaluated the influence of host genetics on gut microbiota in cattle [97], [98], [99], [100], [101] and the possible association of heritable gut microbes with nutrition and gut health in calves [102], methane emissions and feed efficiency in beef and dairy cattle [98], [101]. Majority of these studies used animals belonging to different populations with variable genetic distance, age and diet, thus, masking the real influence of host genetics on gut microbiota. However, a recent study by Fan and colleagues reported genetic influences on gut microbiota based on 228 calves with linearly varying breed (Angus to Brahman), raised under controlled diet and environmental conditions [102]. The three-month-old pre-weaned calves with higher Brahman proportion harboured more butyrate-producing and fibre-digesting bacteria, carbohydrate metabolism genes, less opportunistic pathogenic bacteria and mucin-degraders, lower level of primary antibody (plasma IgG1) and less weight gain than higher Angus proportion calves that harboured bacterial taxa rapidly involved in amino acids and lipids metabolism [102]. This indicates that the host genetics not only shapes the early life gut microbiota composition but can also have strong impact on systemic immunity, which is further associated with health and growth of an animal. However, the studies addressing the role of host genetic influence on neonatal calves’ microbiota are still very scare and needed to be explore further.

## 7 Gut microbiota and the host immune system development

**Fig. 2:**
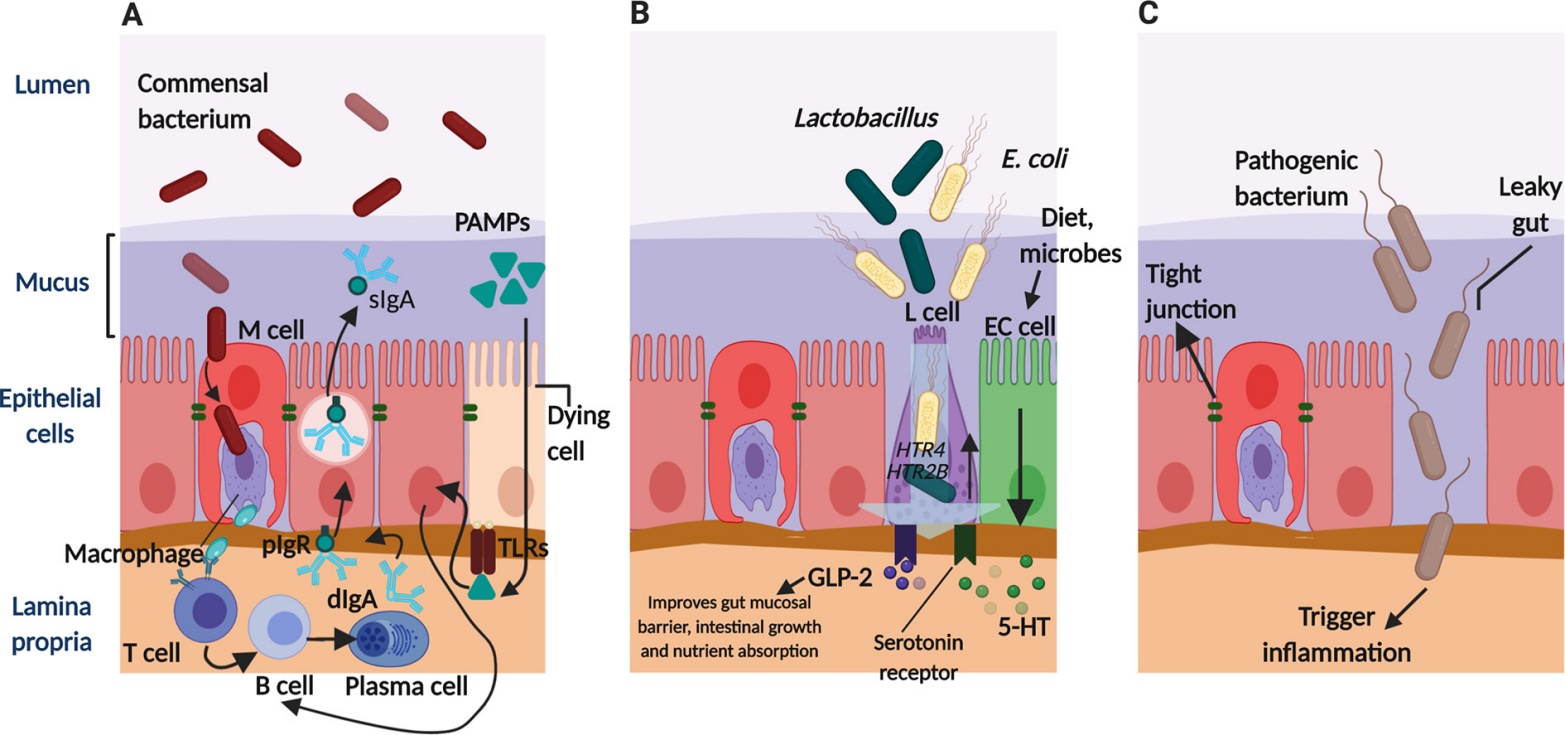
Mucosa-associated lymphoid tissues (MALTs) dependent activation of immune responses in mucosal surface of calves. A) Microfold (M) cell transport microbial antigens from the luminal surface to the underlying MALT cells, where they stimulate specific T- and B- lymphocytes, resulting in the production of dIgA by B-cells, which are translocated as sIgA to the apical epithelial surface. PAMPs can alter the expression of TLRs and activate host immunity. B) Upregulation of *HTR4* and *HTR2B* genes expression by mucosa-associated bacteria. These gene codes for the serotonin receptors that regulate GLP-2 secretion by enteroendocrine L cells via interaction of 5-HT with serotonin receptors. C) Breakdown of tight junctions, transport of pathogens and activation of inflammatory responses. Abbreviations: PAMPs, pathogen-associated molecular patterns; dIgA, dimeric immunoglobulin A; sIgA, secretory immunoglobulin A; pIgR, polymeric Ig receptor; TLRs, toll-like receptors; EC cell, enterochromaffin cell; 5-HT, 5-hydroxytryptamine/serotonin; *HTR4*, 5-hydroxytryptamine receptor 4; *HTR2B,* 5-hydroxytryptamine receptor *2B*; GLP-2, glucagon-like peptide-2. Figure created with BioRender.com.

Gut microbial communities are essential for the development of the mucosal epithelium and immune system of the host [18]. The mucosal epithelial cells line the upper respiratory tract, GIT, and uterus and are the primary responders to the microorganisms [103]. The mucosal immune system contains various physical and chemical barriers as well as pattern recognition receptors (PRRs), which enable the mucosal epithelium to coexist with its resident symbiotic microorganisms and provides protection against invading pathogens [104], [105], [106]. Notably, these signalling cascades are essential for maintaining the intestinal homoeostasis, integrity, antimicrobial peptide expression, and modulation of the mucosal barrier functions and immune responses [91], [107], [108]. The immune response at the mucosal surface is generally initiated by mucosa-associated lymphoid tissues (MALTs) [38], [103]. In ruminants, the initiation of MALT development occurs *in utero* when the microbial communities are not yet established [109]. These *in utero* MALTs are capable of initiating specific immune responses through secretory IgA production [110]. However, IgA+ and IgG+ cells appear in Peyer's patches (PPs) only after birth due to the absence of *in utero* infections [109]. The complete development of germinal centres of PPs requires exposure to the gut microbiome [18]. In the absence of gut microbial exposure, the ileal PPs of new-born lambs showed pre-mature lymphoid follicle involution; however, when the gut microbiome was restored at four weeks, the involution was reversed [111]. This finding demonstrates that the gut microbiome provides signals for the production of a vast variety of pre-immune B cells (Fig. 2A). In addition to the gut microbiome, diet (colostrum, intensive feeding of milk or MR), and environment (toxins) were also found to have a strong influence on the mucosal immune system development in neonatal calves [112]. Extended colostrum feeding during early life resulted in higher abundances of active mucosa-associated *Lactobacillus* and *E. coli* and upregulated the expressions of serotonin and adrenergic receptors genes in the calf’s intestines (Fig. 2B) [113]. These receptors are involved in the regulation of glucagon-like peptide-2 secretion by enteroendocrine L cells, which decreases the apoptosis of epithelial cells, reduces the motility and permeability of the gut, and increases mesenteric blood flow, intestinal growth, and nutrient absorption [114]. A positive correlation was observed between the abundances of *Lactobacillus* and *E. coli* and serotonin receptor gene expression in the colon, suggesting that the early feeding regimen may affect the host–microbe interactions, and thus play a critical role in host immune system development in new-born calves [113]. Likewise, the intensive feeding of milk or MR during the pre-weaning period stimulated the expression of long noncoding RNAs with a potential role in the synthesis of tight junction proteins in the jejunal mucosa of calves [115]. The tight junctions are protective mucosal barriers whose breakdown results in leaky gut syndrome (Fig. 2C) [103], [116]. In addition, it was shown that an ample supply of nutrients is essential for maturation of the intestinal immune system [117], suggesting that the pre-weaning period is critical for the development and maturation of the mucosal immune system in calves [39].

The host identifies commensal microorganisms using PRRs such as toll-like receptors (TLRs) [107]. Mucosa-attached bacteria can also alter the expression of TLRs [118] and cause PRR-dependent activation of the host immunity [14]. In contrast, pathogen-dependent activation of TLR signalling generally activates inflammatory responses [107]. Furthermore, an age-dependent decrease in mucosal TLR gene expression [119] and an increase in T lymphocytes such as CD3+, CD4+, and CD8+ cells in the mucosa of the jejunum and ileum of calves were observed [120]. Such changes may cause a decrease in the innate immunity and an increase in the adaptive immunity with age. This age-dependent downregulation of the innate immunity protects the host from harmful inflammatory responses [121]. It has been suggested that TLRs act as a primary mechanism of innate immunity in neonatal calves. They are substituted by antimicrobial-peptide-dependent innate immune mechanisms over time and protect the animal from unnecessary inflammatory responses [119]. Additionally, a potential link between age-dependent alteration in mucosal immune mechanisms and the gut microbial communities was shown by the negative correlation between TLRs (TLR2, TLR6, and TLR9) in the mucosa of the rumen, jejunum, and caecum and the mucosa-attached bacterial population [119]. Moreover, the host–microbe interactions play a crucial role in the regulation of GIT development, as demonstrated by bovine transcriptome analyses [122].

A positive correlation was observed between the gene copy numbers of *Lactobacillus* or *Bifidobacterium* spp. and microRNAs (miRNA) expression levels. These miRNAs act as promoters of GIT development and include miR-15/16 (immune cells development), miR-29 (maturation of dendritic cells), and miR-196 (lymphoid tissue development) [122]. Likewise, the microbial-driven transcriptional regulation of developing rumen in calves via miRNAs was suggested recently [9]. They identified three miRNA-mRNA pairs involved in the development of rumen “miR-25 and fatty acid-binding protein 7, miR-29a and platelet-derived growth factor α polypeptide, and miR-30 and integrin-linked kinase” [9].

## 8 Role of the microbiota in gut health and treatment strategies

The previous sections have summarised the current knowledge about the essential co-evolution of GIT in ruminants and the colonising microbiome. Disturbances result in an imbalanced symbiosis, leading to gut microbial dysbiosis which can induce several enteric disorders [123]. The pre-weaning period is critical due to the high susceptibility of neonatal calves to a vast variety of bacterial and viral infections, which cause diarrhoea (the major cause of death in neonatal calves) [124]. A decreased incidence of diarrhoea was correlated with a higher abundance of *Faecalibacterium* in faecal samples of one-week-old calves and in the large intestine of three-week-old calves [13], [14], [58]. *F. prausnitzii* promotes anti-inflammatory responses, maintains intestinal homoeostasis [125] and produces butyrate in the large intestine [13]. A high abundance of this species during the pre-weaning period may provide health benefits to the neonates by decreasing their susceptibility to enteric infections. More recently, the idea of a microbiota transplantation to stabilise the gut microbiome was applied in ruminants by transferring the rumen microbiome of adult animals orally to young calves. Although the overall microbiome structure was not affected, the incidence of calf diarrhoea decreased [126].

### 8.1 Early life antimicrobial treatments and emergence of resistant bacterial strains in the calf gut

The dairy industry relies on the use of antimicrobials to cure various diseases, resulting in the production of milk with residual concentrations of antimicrobials [127], [128]. In addition to the presence of antimicrobial residues in the milk, it may contain a high number of pathogens and somatic cells [129]. Thus, the milk from antimicrobial-treated cows is generally used by the dairy industry as a feed for dairy calves [59], [60]. Antimicrobials are also fed directly to the calves as medicated MR to increase their growth rate and prevent diseases [123], [130]. Nevertheless, this direct or indirect exposure of neonatal calves to antimicrobials modifies their intestinal microbial community structure, resulting in the emergence of resistant bacterial strains as well as the transfer of resistance genes to other bacteria [131], [132]. There is increasing evidence of the presence of highly resistant enteric microbes in young animals compared to adults [133], [134], [135], probably due to high faecal–oral transmissions and higher antimicrobial usage in young animals [136]. Several studies have reported the effects of antimicrobial usage on the gut microbial composition [137], [138], [139], [140], [141], [142], the development of antimicrobial-resistant bacterial strains [132], [143], [144], [145], [146], [147], [148], [149], genes involved in antimicrobial resistance [133], [148], [150], and antimicrobial-dependent changes in the functional profile of gut microbiota [151].

Feeding calves with WM containing residual antibiotics (oxytetracycline, ceftiofur, ampicillin, and penicillin) resulted in lower abundances of faecal *Clostridium* and *Streptococcus* in pre-weaned calves [139]. Similarly, when calves were fed with medicated MR containing tetracycline, ceftiofur, penicillin*,* and streptomycin, reduced abundance of *E. coli* in the ileum [140] and *Prevotella* in the rumen [142] was observed. However, feeding calves with MR supplemented with only ceftiofur reduced the abundance of *Comamonas* in the ileum [140]. Decreased abundance of beneficial bacteria (*Faecalibacterium, Roseburia*, *Prevotella,* and *Eubacterium*) and increased abundance of pathogenic bacteria (*Shigella*, *Escherichia*, and *Enterococcus*) in calf faeces were observed using the antibiotic bacitracin methylene disalicylate antibiotics [137]. Enrofloxacin treatment decreased the abundance of *Bacteroides* and increased the abundance of *Blautia*, *Desulfovibrio*, and *Coprococcus* in calf faeces [141]. As the concentration of residual antibiotics in the WM increases, a higher number of antibiotic-resistant bacterial strains emerge in the gut [144]. A higher prevalence of antimicrobial-resistant faecal *E. coli* phenotypes and the increased detection of β-lactamase resistance genes in these populations was observed in WM-fed calves than in bulk milk or MR-fed calves [132], [147], [148]. Feeding drug residues containing milk to the pre-weaned calves also resulted in lower abundance of genes involved in regulation and cell signalling, stress response and nitrogen metabolism [151]. In addition, the direct treatment of calves with antibiotics may also result in the emergence of antibiotic-resistant bacterial strains [149]. However, other studies have reported that the occurrence of multi-drug resistant bacterial strains is not dependent on recent antimicrobial usage but rather on other environmental variables, age, and diet [145], [146], [147]. A decreased prevalence of multi-drug resistant faecal *E. coli* with increasing age of calves indicated that the underdeveloped digestive system of neonatal calves serves as an excellent niche for the growth of resistant microbes due to limited competition for resources [146]**.** However, Thames et al. reported an age-dependent increase in tetracycline resistance genes in calf faeces [150]. These studies suggest that the direct and indirect exposure of the gut of neonatal calves to the antimicrobials modifies the composition and functional profile of the microbiome and the development of antibiotic resistance is mainly influenced by host-specific factors.

### 8.2 Improvement of calf gut health by feed supplements

The use of antimicrobials to support calves’ health and to prevent or treat certain diseases can be avoided by using direct-fed microbes, prebiotics, and probiotics. This has been widely practised in order to improve gut health and productivity of livestock [152], [153]. Supplementation of new-born calves with *Lactobacillus* and *Bifidobacterium* within the first seven days of life decreased diarrhoea and increased feed conversion ratio and weight gain [154]. Similarly, supplementation with *F. prausnitzii* in the first week of calf life decreased the calf death rate and diarrhoea [155]. Administration of *Lactobacillus* spp. to young calves also increased their serum IgG levels, suggesting a potential role of the host–microbe interactions in modulating calf health [156]. Apart from influencing host health, microbial manipulations also affect the gut microbial community structure. Feeding pre-weaned calves with probiotic strains decreased their intestinal colonisation with pathogenic *E. coli* [157]. Similarly, a decrease in faecal *E. coli* load was observed using direct-fed microbes [158]. Supplementation of the diet of neonatal calves with *Saccharomyces cerevisiae boulardii* immediately after birth increased the abundance of beneficial bacteria (*F. prausnitzii* and *Lactobacillus*) in the intestinal microbiota, as well as increasing the concentrations of endogenous secretory IgA, thus enhancing immunity and intestinal homoeostasis of calf GIT [159]. Feeding heated colostrum soon after birth benefited young calves with increased colonisation with *Bifidobacterium* and decreased colonisation with *E. coli* in the small intestine, suggesting the potential role of colostrum as a natural prebiotic associated with reduced risk of diarrhoea [41], [53]. Prebiotics supplementation immediately after birth was found to have more prominent effects than supplementation at a later stage. Higher abundances of *Bifidobacterium* and *Lactobacillus* were detected in the colon of two-week-old than in four-week-old calves fed with galactooligosaccharides [160]. Supplementation of grazing calf diet with cellooligosaccharides decreased the proportions of archaea at weaning and *Fibrobacter* within the first four weeks post-weaning. In contrast, an increase in *Fibrobacter* was detected using kraft pulp as prebiotics at four weeks post-weaning [161]. Addition of astragalus root extract in the diet of early weaned calves at a dose of 2% dry matter intake, increased the body weight, average daily gain, serum concentrations of interleukin-2 (IL-2), IgG, superoxide dismutase, and the abundance of fibrolytic bacteria [162]. Increasing the dose of astragalus root extract to 5% and 8% dry matter intake fortified these effects [162]. Supplementation of calf diet with zinc oxide (104 mg/d) effectively reduced the incidence of diarrhoea from days 1–3, increased the abundance of beneficial *Faecalibacterium* and *Lactobacillus* within the first seven days of life and improved the immunity by increasing the concentrations of serum immunoglobulins (IgM and IgG) [163]. However, when zinc methionine (457 mg/d) was supplemented, a prolonged reduction in diarrhoea was observed from days 1–14, and increased abundances of *Faecalibacterium* and *Collinsella* (day 7), and *Ruminococcus* (2 weeks) were detected [163]. These results suggest the essential role of zinc in the treatment of neonatal calf diarrhoea. In addition, calcium propionate supplementation increased the body weight and decreased the relative abundance of Bacteroidetes in both pre- and post-weaning groups, but increased Proteobacteria (*Succinivibrionaceae*) and *Methanobrevibacter* only the post-weaning group [164]. These studies suggest that microbial manipulations are easier to perform during early life, and these effects may persist longer when manipulations are performed in early life of animal.

## 9 Summary and outlook

**Fig. 3:**
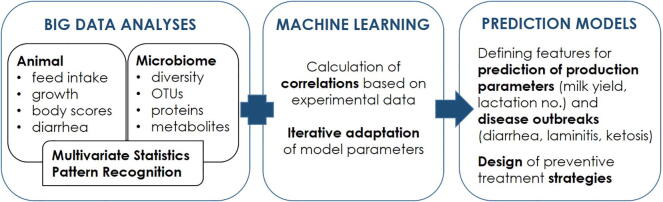
Combination of big data repositories with machine learning algorithms to create prediction tools for sustainable animal productions strategies.

Understanding the pattern of microbial succession throughout the GIT of pre-weaned calves is essential as it influences the development and maturation of the host gut, immune system, and health. The microbial colonisation of the GIT of neonatal calves begins during the birthing process or even *in utero*, but the microbial community structure changes rapidly within the first few weeks of life and is strongly affected by the genetic background, rearing environment, early life antibiotic treatments, age and feeding conditions. The majority of the studies reported the early life microbial succession patterns using DNA-based methods without any information about the viability, genetic potential (metagenomics), or even gene or protein expression (metatranscriptomics and metaproteomics) of the detected microbial communities. Thus, there is still a lot to understand the underlying mechanisms of the possible interactions between the gut microbial communities and their mammalian host. In addition, the results obtained by various DNA-based studies are limited by different sample types and locations, extraction methods, gene regions being sequenced, sequencing methods, sequence depth, and the pipeline used for the analysis. In addition to the region-specific establishment of microbial communities along the GIT of calves, the microbiota associated with the epithelial mucosa was clearly different from those occupying the luminal digesta and had a potential role in host immune system development [113]. Thus, to better understand the host–microbe interactions, a thorough knowledge of microbial segregation between mucosal epithelium and luminal digesta throughout the pre-weaning period is of utmost importance. In the future, genome-wide association studies should be conducted to track the possible associations between host single nucleotide polymorphisms and the abundances of commensal bacterial taxa. Furthermore, more emphasis should be placed on the microbial dysbiosis caused by in-feed antimicrobials and the possibility of using the gut microbiome, prebiotics, and probiotics as antimicrobial substitutes. In addition to the control of neonatal calf diseases using antimicrobial alternatives, one can also predict the onset of diseases based on early life gut microbiota composition, and the predictive modelling approach was recently suggested by Ma et al. [165]. The combination of collecting big data with machine learning algorithms can support the establishment of prediction tools for output targets or disease outbreaks, and helps to design preventive treatment strategies (Fig. 3). We conclude by mentioning that future studies must focus on the ecologic as well as metabolic activity of the detected microbiome based on advanced machine learning and prediction modelling approaches.

## Declaration of Competing Interest

The authors declare that they have no known competing financial interests or personal relationships that could have appeared to influence the work reported in this paper.
